# Contraceptive use among adolescent girls and young women ages 15–24 in seven high HIV prevalence countries

**DOI:** 10.3389/frph.2025.1667613

**Published:** 2025-11-11

**Authors:** Abigail R. Greenleaf, Karam Sachathep, Emma Geisler, Tara F. Abularrage, Giles A. Reid, Felix Ndagije, Tepa Nkumbula, Harriet Nuwagaba-Biribonwoha, Elaine Abrams, Neena M. Philip

**Affiliations:** 1 ICAP at Columbia University, New York, NY, United States; 2 Department of Population and Family Health, Mailman School of Public Health, Columbia University, New York, NY, United States; 3 ICAP at Columbia University, Maseru, Lesotho; 4 ICAP at Columbia University, Lusaka, Zambia; 5 ICAP at Columbia University, Mbabane, Eswatini; 6 Department of Epidemiology, Mailman School of Public Health, Columbia University, New York, NY, United States; 7 Department of Pediatrics, Vagelos College of Physicians & Surgeons, Columbia University, New York, NY, United States

**Keywords:** contraception, HIV, adolescent girls and young women, Sub-Saharan Africa, survey

## Abstract

**Background:**

Adolescent Girls and Young Women (AGYW; ages 15–24) continue to use contraceptives at lower rates than older women in sub-Saharan Africa. We describe contraceptive use among AGYW in seven Southern African countries (Botswana, Eswatini, Lesotho, Malawi, Mozambique, Zambia and Zimbabwe).

**Methods:**

Cross-sectional, nationally representative household-based data from seven Population-based HIV Impact Assessment surveys (conducted between November 2019 and February 2022) were analyzed using survey weights to create descriptive results and pooled odds of modern contraceptive use.

**Results:**

Among the 11,094 AGYW, contraceptive use (male or female sterilization, IUD, implants, injectables, pills, condoms) ranged from 45.0% in Mozambique to 75.1% in Botswana. Condoms were the most frequently reported method in four of seven countries (Botswana 61% of those using modern methods use condoms, Eswatini 66%, Lesotho 49% and Mozambique 33%). Dual method (use of any modern contraceptive method plus a condom) ranged from <1% in Malawi to 15% in Botswana. When conducting a pooled multivariable logistic regression, higher odds of modern contraceptive use was associated with higher education [Odds Ratio (OR) 1.7, 95% Confidence Interval (CI) 1.5–2.0], being in the highest wealth quintile (OR 1.5, 95% CI 1.2- 2.0), and having children (one birth: OR 2.0 95% CI 1.7–2.4), two or more: (2.5, 95% CI 2.0–3.0), but was lower among AGYW living with HIV (OR 0.7 95% CI 0.6–0.9).

**Conclusions:**

Contraceptive prevalence rates varied by country but across countries, AGYW in Southern Africa commonly use short-acting methods, and specifically condoms: a user-dependent method prone to inconsistent use. Efforts to expand access to diverse, youth-friendly contraceptive options - particularly short-acting and multipurpose methods - could better align with the needs of AGYW. These findings can inform policies and programs aiming to reduce unmet contraceptive need and improve reproductive health outcomes among AGYW in the region.

## Introduction

Unmet need for family planning remains high in sub-Saharan Africa (SSA); the region with the lowest modern contraceptive prevalence rates (23.6%), and the lowest regional average contraceptive demand satisfied (52.0% in 2019) (1). Adolescent girls and young women (AGYW; ages 15–24) in particular remain a priority population for increasing contraceptive use in SSA (2). However, while investments in young women's health benefit current adolescents, their futures, and the next generation, improvements in adolescent contraceptive use have lagged as compared with older women (3, 4). A combination of social, economic, and systemic barriers lead to low contraceptive use among AGYW compared to older women, with stigma, restricted access, and lack of empowerment standing out as key challenges (5).

In Southern Africa, AGYW have higher levels of contraceptive use compared to AGYW in other regions in SSA, which also translates to the lowest regional total fertility rate (TFR 3.2) in SSA (6). However, in at least one instance in Southern Africa, unmet need for family planning recently increased: in Eswatini, unmet need among those 20–24 aged increased from 17.4% in 2014 to 30.3% in 2021 (7). AGYW in Southern Africa are also highly impacted by HIV: Southern Africa accounts for one-third of the global HIV burden (8) and the impact is greater among AGYW, who are twice as likely to be living with HIV (9) and three times as likely to be newly infected with HIV (10) compared with adolescent boys and young men of the same age.

Condoms are a core component of HIV prevention efforts, as they offer dual protection against sexually transmitted infections (including HIV) and unintended pregnancy and have been strongly promoted as part of international initiatives to address the HIV/AIDS epidemic. Condom use is higher among AGYW, who use short-acting methods (condoms and pills) more often than older women (11). Unmarried women have sex less frequently than married women, and infrequent sex is associated with not using long-acting contraceptive use (12). However, condoms – which are fairly accessible (i.e., available outside of health settings) in Southern Africa – are less effective than other modern family planning methods that are not coital-specific.

To align with World Health Organization's guidelines on ensuring human rights in the provision of contraceptive information and services (13) and in particular, accountability in the delivery of contraceptive information and services – we describe modern contraceptive use among AGYW in seven high HIV-burden Southern African countries: Botswana, Eswatini, Lesotho, Malawi, Mozambique, Zambia and Zimbabwe. We estimate modern contraceptive prevalence rates, detail the mix of contraceptive methods used, and identify characteristics associated with modern contraceptive use.

## Materials and methods

### Data source

Population-based HIV Impact Assessment (PHIA) surveys, which aimed to obtain nationally representative measures of HIV incidence and prevalence as well as evaluate the status of national HIV programs, were conducted in Botswana, Eswatini, Lesotho, Malawi, Mozambique, Zambia, and Zimbabwe between November 2019 and February 2022. Interviewers administered questionnaires in participant homes and trained medical staff conducted HIV testing. Thus all data was self-reported except HIV status. Consent or assent was obtained from heads of households and individuals aged 15 and above (except in Botswana, where participants included were aged 15–64 years). Assent from parents or guardians was obtained for minors aged 15–17 years. All household response rates were above 83%. Detailed information on study design, sampling and response rates are available elsewhere (14–17).

### Sample

All females ages 15–24 who were not pregnant and reported having sex in the past 12 months and answered the question “Are you or your partner currently doing anything to avoid or delay getting pregnant?” (our main outcome of interest) were included in this analysis. We also excluded any participants who reported using more than two modern methods that cannot feasibly be used together (e.g., IUD and implant; *n* = 19).

### Measures

Those included in our analysis were considered a modern user if they self-reported any of the following methods: male or female condoms, injections, pill, female or male sterilization, IUD or implant. If a woman reported currently using more than one method, she was classified as a user of the most effective method. We estimated modern contraceptive prevalence by including anyone who reported a modern method in the numerator and all women in our sample in the denominator. We also estimate three groups of contraceptive users: non-users (reported no modern methods); condom-users only (i.e., only method reported was condoms); modern users minus condoms (using a modern method other than condoms). Those who reported a modern method and a male or female condom were considered a dual user. We also estimate contraceptive method mix: the denominator is women using a modern method, and the numerator is divided by methods to show the distribution of method types among users in a population. This indicator reflects both supply factors (such as the availability and affordability of methods) and demand factors (such as client preferences) (18).

Socio-demographic variables included residence (urban or rural; Lesotho was the sole country with a third classification – peri-urban. This group was merged with urban dwellers); wealth quintiles (created via household assets listing and primary components analysis); and self-reported age; education (no formal school, completed primary, completed secondary, completed more than secondary); parity (0, 1, or 2 or more live births); marital status (not currently married or currently married or living together); HIV status (living with HIV or HIV negative; confirmed by test during the PHIA survey). Additional details on item-level missingness (minimal) HIV testing and overall survey procedures are available elsewhere (14).

### Analysis

We analyzed the de-identified secondary data using STATA version 18.0. PHIA survey weights accounted for survey design, non-response rates, and post-stratification. All counts were unweighted. Country-specific point estimates were weighted, with standard error derived using Jackknife replicates. To compare between countries, we first conducted country-specific analysis, describing the survey population by country as well as modern contraceptive prevalence rate and method mix. We then included a pooled description of the population to describe regional trends and create a larger sample size. Combined jackknife replicate weights were used for pooled variance estimation. These were derived by expanding the replicate weight array for each country to match the country with the highest number of replicates, then placing the replicate weights in a random order and filling blanks with the base weight. After stacking together the resulting country datasets, pooled estimates were derived using these weights account for the relative populations and complex sample designs of each country. For the regressions, we used the pooled dataset and the aforementioned replicated weights to conduct bi-variate then multivariable logistic regression.

### Ethical approval

Ethical approval was obtained from each local Institutional Review Board (see 45 C.F.R. part 46; 21 C.F.R. part 56.), Centers for Disease Control and Prevention, and Columbia University (Eswatini, Lesotho, Malawi, Mozambique and Zimbabwe) or University of Maryland – Baltimore (Botswana and Zambia).

## Results

A total of 11,094 AGYW were included in this analysis; by country: Botswana (1,184), Eswatini (907), Lesotho (1,435), Malawi (2,420), Mozambique (1,643), Zambia (1,965), Zimbabwe (1,540) (Table 1). The pooled mean age was 20.2 years (SE 2.5), 93.3% had primary education or higher, and 52.0% were married. Overall, 35.8% were nulliparous, 38.7% reported one live birth and 25.4% reported two or more live births. Seven (6.8%) percent were living with HIV.

**Table 1:** Characteristics of adolescent girls and young women (15–24) in seven Southern Africa countries (2019–2022).

Country	Botswana	Eswatini	Lesotho	Malawi	Mozambique	Zambia	Zimbabwe	Pooled total
Number of AGYW	*N* = 1,184	*N* = 907	*N* = 1,435	*N* = 2,420	*N* = 1,643	*N* = 1,965	*N* = 1,540	*N* = 11,094
% of total sample	11%	8%	13%	22%	15%	18%	14%	100%
Age in years	21.1 (2.2)	20.6 (2.4)	20.6 (2.4)	20.2 (2.5)	19.9 (2.4)	20.3 (2.6)	20.7 (2.3)	20.2 (2.5)
Residence								
Rural	516 (32.2%)	716 (66.2%)	684 (43.9%)	1,998 (82.6%)	841 (54.9%)	1,222 (59.4%)	1,048 (63.9%)	7,025 (62.2%)
Urban	668 (67.8%)	191 (33.8%)	751 (56.1%)	422 (17.4%)	802 (45.1%)	743 (40.6%)	492 (36.1%)	4,069 (37.8%)
Highest school attended								
No education	14 (1.2%)	9 (0.9%)	14 (0.9%)	77 (3.4%)	170 (12.2%)	93 (4.5%)	14 (1.0%)	391 (6.7%)
Primary	32 (2.2%)	101 (11.1%)	285 (18.5%)	1,628 (66.8%)	651 (42.2%)	667 (33.0%)	397 (23.7%)	3,761 (41.2%)
Secondary	889 (70.0%)	698 (74.3%)	987 (69.2%)	666 (27.6%)	785 (43.6%)	1,152 (58.5%)	1,067 (70.7%)	6,244 (48.5%)
More than secondary	249 (26.6%)	99 (13.7%)	149 (11.5%)	49 (2.1%)	33 (1.8%)	53 (3.9%)	62 (4.6%)	694 (3.5%)
Wealth quintiles								
Lowest	317 (18.3%)	229 (21.6%)	333 (20.5%)	484 (20.4%)	178 (12.6%)	594 (23.1%)	403 (22.0%)	2,538 (18.0%)
Second	276 (22.7%)	262 (25.4%)	273 (18.4%)	541 (22.1%)	245 (15.7%)	401 (19.2%)	296 (17.8%)	2,294 (18.4%)
Middle	221 (20.6%)	163 (17.5%)	292 (20.7%)	484 (19.6%)	266 (17.0%)	323 (19.2%)	274 (18.5%)	2,023 (18.4%)
Fourth	214 (22.7%)	145 (19.8%)	320 (24.3%)	444 (18.0%)	428 (27.4%)	340 (19.7%)	277 (21.9%)	2,168 (22.9%)
Highest	156 (15.7%)	108 (15.7%)	197 (16.2%)	467 (19.9%)	521 (27.2%)	307 (18.8%)	290 (19.8%)	2,046 (22.4%)
Marital status								
Not married	978 (83.5%)	775 (87.4%)	779 (56.8%)	964 (42.9%)	820 (49.1%)	1,013 (52.9%)	557 (35.6%)	5,886 (48.0%)
Married	202 (16.5%)	127 (12.6%)	654 (43.2%)	1,455 (57.1%)	821 (50.9%)	951 (47.1%)	983 (64.4%)	5,193 (52.0%)
Number of live births								
None	576 (50.2%)	398 (47.6%)	666 (49.0%)	596 (28.1%)	698 (42.1%)	605 (32.6%)	422 (28.8%)	3,961 (35.8%)
One	436 (37.3%)	362 (38.1%)	618 (41.3%)	1,096 (44.6%)	517 (31.1%)	810 (40.9%)	751 (48.6%)	4,590 (38.7%)
Two or more	170 (12.5%)	141 (14.3%)	148 (9.6%)	728 (27.3%)	428 (26.8%)	549 (26.5%)	366 (22.6%)	2,530 (25.4%)
HIV Status								
HIV Negative	1,109 (94.9%)	789 (86.7%)	1,278 (89.1%)	2,318 (96.6%)	1,494 (90.1%)	1,867 (95.9%)	1,440 (94.2%)	10,295 (93.2%)
Living with HIV	75 (5.1%)	118 (13.3%)	157 (10.9%)	102 (3.4%)	149 (9.9%)	98 (4.1%)	100 (5.8%)	799 (6.8%)

**Figure 1:**
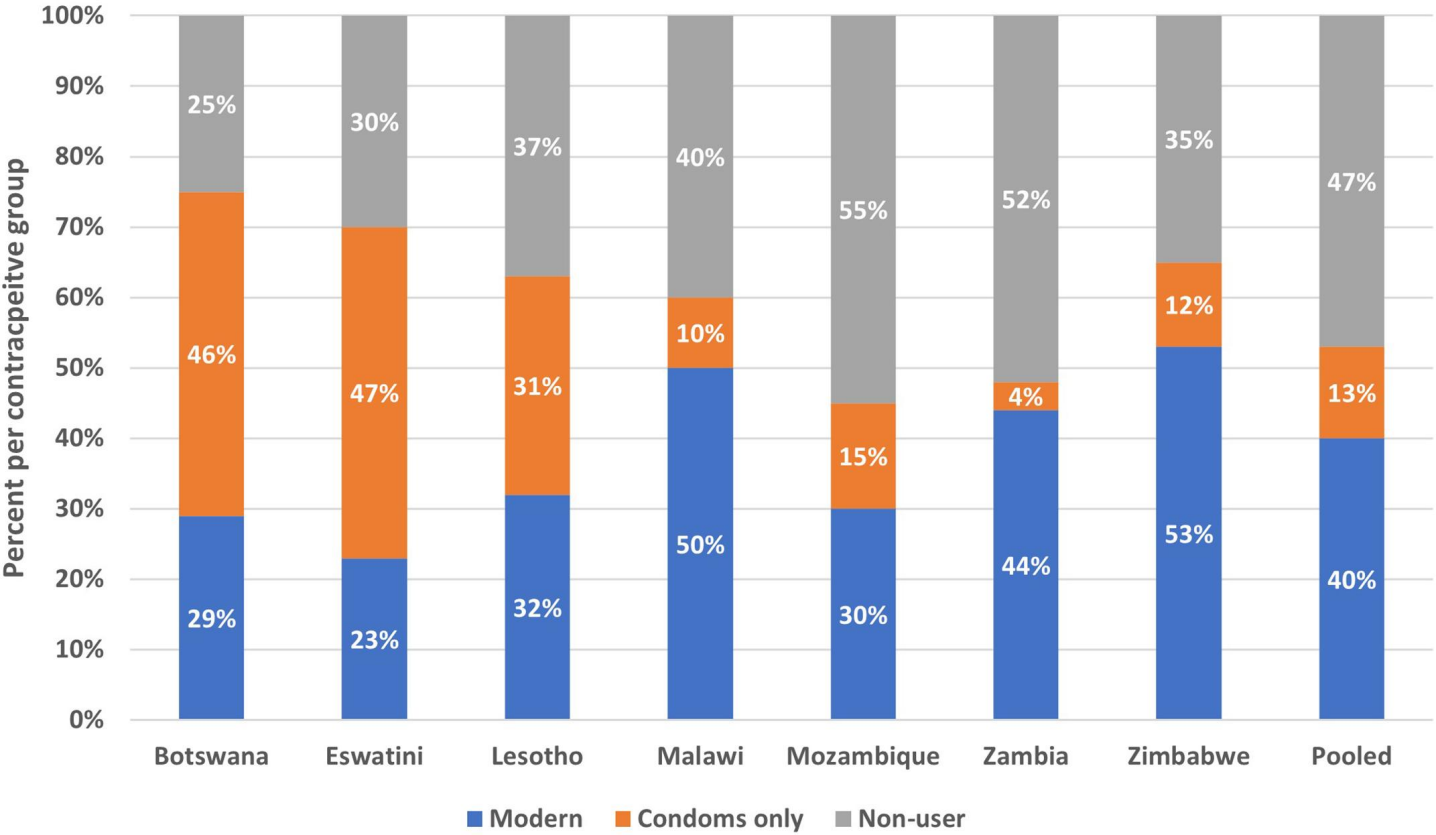
Percent of AGYW by country that are using a modern method other than condoms, percent using condoms only, and percent non-users (2019–2022).

Modern contraceptive prevalence rates (mCPR) among AGYW were highest in Botswana (75.1%) followed by Eswatini (70.3%), Zimbabwe (65.0%), Lesotho (63.0%), Malawi (60.1%), Zambia (48.0%) then Mozambique (45.0%). In two countries (Botswana and Eswatini) a larger proportion of women used condoms than all other modern methods combined (Figure 1).

Four methods: condom, pill, injection and implant constituted more than 97% of the modern method mix in all countries. Condoms were among the three most common modern methods in all countries except Zambia (Table 2). In four of the seven countries, condoms were the most frequently reported method (Botswana 61%, Eswatini 66%, Lesotho 49% and Mozambique 33% of modern contraceptive users). In Zimbabwe condoms were the second most frequent (19%), in Malawi third (16%) and in Zambia fourth (8%). The pill was most common in Zimbabwe, (52%). In Malawi and Zambia, injection was most common (47%, 57%). There was method skew (a single method accounting for more than 50% of all use) (18) in four countries: Botswana and Eswatini (condoms), Zambia (injection) and Zimbabwe (pill).

**Table 2:** Modern contraceptive prevalence rate (mCPR) and method Mix among users by country among adolescent girls and young women, 2019-2022.

Country	Botswana	Eswatini	Lesotho	Malawi	Mozambique	Zimbabwe	Zambia	Pooled
mCPR 95% CI	75% (71%–79%)	70% (67%–73%)	63% (60%–66%)	60% (58%–62%)	45% (42%–48%)	65% (62%–68%)	48% (45%–51%)	53% (52%–54%)
Method mix among modern users (% using each method)								
Condom	61	66	49	16	33	19	8	24
Pill	10	18	10	3	14	52	12	17
Injection	13	9	34	47	27	15	57	34
Implant	12	5	4	30	24	13	22	22
IUD	1	0	1	2	1	2	1	2
Other methods^a^	3	2	2	2	1	0	0	1
	Most used	2nd most used	3rd most used					

a Includes female or male sterilization, withdrawal, beads.

The prevalence of dual method use (i.e., condoms plus a second modern method) was low among AGYW, with 16.1% reporting dual method use in Botswana, followed by Lesotho (8.6%), Mozambique (7.6%), Eswatini (5.6%), Zambia (2.5%), Zimbabwe (2.0%) and less than one percent in Malawi.

When conducting a pooled multivariable logistic regression, higher odds of modern contraceptive use was associated with higher education, more wealth, and parity, but was lower among AGYW living with HIV (Table 3). Specifically, compared to those with no or primary education only, those with secondary or higher had 1.7 [Odds Ratio (OR) 95% Confidence Interval (CI) 1.5–2.0] higher likelihood of modern contraceptive use. Only those in the highest wealth quintile had higher likelihood of modern contraceptive use (OR 1.5, 95% CI 1.2–2.0) compared to those in the lowest quintile. Compared to Botswana, Lesotho (OR 0.6, 95% CI 0.5–0.8), Malawi (OR 0.6, 95% CI 0.5–0.8), Mozambique (OR 0.3, 95% CI 0.2–0.4), Zambia (OR 0.3, 95% CI 0.2–0.4) and Zimbabwe (OR 0.6, 95% CI 0.4–0.7) had lower likelihood of modern contraceptive use. Compared to nulliparous AGYW, the odds of modern contraceptive use were 2.0 (95% CI 1.8–2.4) and 2.5 (95% CI 2.1–3.1) times greater among AGYW with one and two or more births, respectively. Finally, those living with HIV had lower likelihood of modern contraceptive use compared to those who were not living with HIV (OR 0.7, 95% CI 0.5–0.9).

**Table 3:** Pooled odds of using modern contraception among adolescent girls and young women, 2019–2021.

	Bi-Variate			Multi-variable		
	Odds ratio	Lower 95% CI	Upper 95% CI	Odds ratio	Lower 95% CI	Upper 95% CI
Age (Reference: Ages 15–19)						
20–24	1.7	1.5	1.9	1.2	1.0	1.4
Geography (Reference: Rural)						
Urban	1.3	1.2	1.5	1.2	1.0	1.5
Education Level (Reference: Primary or less)						
Secondary or more	1.6	1.5	1.8	1.7	1.5	2.0
Wealth Quintile (Reference: Lowest Quintile)						
Second	1.0	0.8	1.2	1.0	0.8	1.2
Middle	1.0	0.8	1.2	1.0	0.8	1.2
Fourth	1.2	1.0	1.4	1.2	0.9	1.4
Highest	1.4	1.2	1.7	1.5	1.2	2.0
Country (Reference: Botswana)						
Eswatini	0.8	0.6	1.0	0.9	0.7	1.2
Lesotho	0.6	0.5	0.7	0.6	0.5	0.8
Malawi	0.5	0.4	0.6	0.6	0.5	0.8
Mozambique	0.3	0.2	0.3	0.3	0.2	0.4
Zambia	0.4	0.2	0.4	0.3	0.2	0.4
Zimbabwe	0.6	0.5	0.8	0.6	0.4	0.7
Parity (Reference: No children)						
One birth	1.9	1.7	2.1	2.0	1.7	2.4
Two or more births	1.9	1.7	2.2	2.5	2.0	3.0
Marital Status (Reference: Unmarried)						
Married	1.3	1.2	1.5	1.2	1.0	1.3
HIV Status (Reference: Negative)						
Living with HIV	0.7	0.6	0.9	0.7	0.6	0.9

## Discussion

Modern contraceptive prevalence rates among AGYW were above 50% in five of the seven countries. The pooled mCPR estimate among AGYW is 53%, a similar finding to other regional analyses among women of reproductive age in SSA indicating mCPR of 50.1% and unmet contraceptive need of 18.1% (19). Method skew is either present (Botswana, Eswatini, Zimbabwe, Zambia) or is verging (Malawi, Lesotho) across six of the seven analyzed countries. The demonstrated frequency of method skew particularly towards short-cting methods, when combined with regional estimates of unmet contraceptive need, illustrate the potential value of increased access to a diverse method mix, to better meet the family planning needs of AGYW in these settings.

When examining method mix, the dependence on condoms – a coital-specific method – was high, with condoms being the most used method in four countries. In fact, if those who only reported condom use were considered non-users, the mCPR would substantially decrease in Botswana (75% to 28%), Eswatini (70%–24%), and Lesotho (63%–32%), the countries with the most severe HIV epidemics. Most users did not report dual method use. Report of condom use is not well captured in surveys and report of use differs by marital status recency of sexual activity (12, 20). Condoms are easily accessible and require minimal planning, making them a useful tool for young people who are not having sex frequently and thus lack motivation to use a longer-acting method. However, the heavy dependence on condoms puts young women at higher risk of unintended pregnancy given consistent condom use is proven difficult (21). Therefore, in countries with high reliance on condoms, it may be more meaningful to include condom use as a complimentary measure to mCPR instead of part of mCPR. Joint efforts between national HIV and reproductive health programs could be an important avenue towards diversifying the contraceptive method mix for AGYW in SSA. Various strategies could be considered including community-level champions to facilitate demand for diverse method access and uptake. Providing accurate, person-centered information through provider counseling, peer-groups, social media, and digital technology can also help potential AGYW clients to become better informed to meet their personal family planning needs (22).

The characteristics associated with modern contraceptive use among our population of AGYW in seven southern African countries (higher education, more wealth, parity) align with characteristics associated with modern contraceptive use in SSA (23, 24). While in many contexts married women are less likely to use contraception than unmarried women (25), the trend of sexual debut for young women before marriage (given age at marriage is increasing) is more pronounced in Southern Africa compared to other regions of SSA, and may be reflected in our results (26, 27). However, we found the odds of modern contraceptive use were lower among those living with HIV compared to those who were HIV-negative. Previously published multi-country analyses of PHIA data found that among all women of reproductive age, women with HIV were more likely to report contraceptive use (28) but our focus on AGYW yielded different results. Reasons for lower contraceptive use among AGYW living with HIV should be explored qualitatively and quantitatively.

This study makes several important contributions. The study presents data on the prevalence of modern contraceptive use among a population at high risk for undesired pregnancy, emphasizing the current state of family planning efforts in the region. Moreover, it provides insights into the method mix among AGYW in Southern Africa, highlighting the heavy reliance on condoms in over half the countries. It also identifies the key determinants of contraceptive use across the population, which align with other literature from across SSA. The pooled results increased the sample size and found AGYW living with HIV had lower odds of modern contraceptive use compared to HIV-negative AGYW, which was not apparent in country-specific analysis. Overall, the findings highlight differences by country and can inform policy and programmatic interventions aimed at improving contraceptive access and uptake among AGYW in Southern Africa. Given sexual activity is often sporadic among young people, they may prefer coital-specific methods over long-acting methods. Infrequent sex may demotivate long-acting contraceptive use thus biomedical research should not abandon development of improved or new short-acting options. For example, emergency contraception is used frequently among young people in Eswatini (29).

Our results are important to consider in the context of increasing PrEP availability. Whether PrEP uptake decreases condom use has mixed findings: among a different population (men who have sex with men), a systematic review found increased condomless sex among PrEP users (30) but other studies have found no change or even increased use (31, 32). If condom use were to decrease with PrEP uptake and public health officials do not proactively address this shift in methods, the mCPR may decrease and unmet need could increase for other methods; particularly given dual method use is <16% in all countries. Particular attention should be paid to Eswatini and Botswana, where we found a notable method skew (19) in favor of condoms. It is unknown if the skew towards condoms is demand or supply-driven, but this should be explored to better prepare method availability as PrEP continues to become more common and if condom use decreases.

A primary strength of this analysis is that it uses multi-country data, allowing a comparison of contraceptive use patterns across Southern Africa. Additionally, the datasets are nationally representative, enhancing the generalizability of the findings. However, country-specific variations may be masked when polling the data and the study is limited by its cross-sectional design, which restricts the ability to infer causality or observe changes over time. Also, the PHIAs did not ask questions to calculate unmet need for contraception nor did PHIA ask about emergency contraception; information about both would have helped better understand contraceptive use.

In summary, about half of AGYW in Southern Africa are using modern contraception, many of whom are using condoms, a coital-specific method that is non-private, user-dependent and prone to higher typical-use failure rates, thus increasing risk of an unintended pregnancy. Higher education, more wealth and parity were associated with modern contraceptive use while living with HIV decreased the odds. Given most AGYW report infrequent sex, promoting and ensuring access to short-acting methods; including multi-purpose technologies currently in trial (33)– rather than long-acting methods that young people may not be motivated to adopt – could offer more effective HIV protection and contraception for this population.

## Data Availability

Publicly available datasets were analyzed in this study. This data can be found here: https://phia-data.icap.columbia.edu/datasets.

## Appendix 1 Adjusted Odds of Using Modern Contraception among Adolescent Girls and Young Women, 2019–2021 by Country

**Table:**

	Botswana	Eswatini	Lesotho	Malawi	Mozambique	Zambia	Zimbabwe
Number of AGYW	*N* = 1,178	*N* = 889	*N* = 730	*N* = 2,419	*N* = 1,632	*N* = 1,963	*N* = 1,539
% of total sample	11%	8%	13%	22%	15%	18%	14%
Age (Ref: Ages 15–19)							
20–24	0.9 (0.5–1.5)	2.1 (1.4–3.0)	1.2 (0.8–1.8)	1.1 (0.8–1.3)	1.2 (0.9–1.5)	1.3 (1.0–1.8)	1.3 (1.0–1.7)
Residence (Ref: Rural)							
Urban	0.8 (0.5–1.1)	1.1 (0.7–1.7)	1.0 (0.6–1.4)	1.1 (0.8–1.5)	0.9 (0.6–1.2)	2.3 (1.5–3.4)	2.6 (1.6–4.2)
Highest school attended (Ref: None)							
Primary	0.7 (0.1–5.6)	0.9 (0.4–1.8)	1.1 (0.5–2.1)	1.1 (0.6–1.8)	1.2 (0.7–1.8)	1.0 (0.5–1.7)	3.3 (1.1–9.9)
Secondary	0.9 (0.2–5.3)	1.1 (0.6–2.0)	1.0 (0.6–1.5)	1.3 (0.7–2.2)	2.5 (1.5–4.0)	1.1 (0.6–1.9)	3.0 (1.1–9.9)
More than secondary	0.8 (0.1–4.6)			0.6 (0.2–1.5)	17.8 (3.8–84.0)	1.3 (0.5–3.5)	4.6 (1.3–16.1)
Wealth quintiles (Ref: Lowest)							
Second	0.5 (0.3–0.9)	1.1 (0.7–1.6)	0.3 (0.1–1.5)	1.2 (0.9–1.6)	1.4 (0.8–2.3)	0.8 (9.6–1.1)	1.0 (0.7–1.4)
Middle	0.4 (0.3–0.8)	1.0 (0.6–1.6)	0.4 (0.1–1.9)	1.0 (0.7–1.3)	1.5 (0.9–2.6)	1.0 (0.6–1.5)	1.1 (0.7–1.5)
Fourth	0.5 (0.3–0.9)	1.0 (0.6–1.6)	0.4 (0.1–1.9)	1.0 (0.7–1.3)	2.8 (1.7–4.8)	0.7 (0.4–1.2)	0.5 (0.3–0.9)
Highest	0.3 (01–0.6)	0.9 (0.5–1.5)	0.5 (0.1–2.6)	0.8 (0.6–1.2)	4.3 (2.5–7.7)	0.5 (0.3–0.9)	0.7 (0.4–1.2)
Marital status (Ref: Unmarried)							
Married	1.6 (0.9–2.9)	0.9 (0.6–1.5)	1.1 (0.7–1.7)	1.9 (1.6–2.4)	0.7 (0.5–0.9)	1.6 (1.2–2.1)	1.5 (1.1–2.0)
Number of live births (Ref: 0)							
One	0.9 (0.5–1.4)	0.8 (0.5–1.1)	1.9 (1.2–2.8)	2.6 (2.0–3.3)	1.1 (0.8–1.5)	4.9 (3.5–6.7)	4.4 (3.2–6.0)
Two or more	1.3 (0.7–2.5)	0.6 (0.4–1.1)	5.1 (2.2–12.1)	2.8 (2.0–3.8)	1.5 (1.0–2.2)	7.0 (4.6–10.6)	7.7 (5.0–11.4)
HIV Status (Ref: Negative)							
Living with HIV	1.7 (0.8–3.8)	1.0 (0.6–1.6)	0.8 (0.5–1.2)	1.0 (0.7–1.5)	0.6 (0.4–0.9)	1.1 (0.6 (1.9)	0.9 (0.5–1.5)
